# The nest fungus of the lower termite *Reticulitermes labralis*

**DOI:** 10.1038/s41598-019-40229-x

**Published:** 2019-03-04

**Authors:** Chenxu Ye, Jing Li, Yuehua Ran, Humaira Rasheed, Lianxi Xing, Xiaohong Su

**Affiliations:** 1 Key Laboratory of Resource Biology and Biotechnology in Western China (Northwest University), Ministry of Education, Xi’an, China; 2 Shaanxi Key Laboratory for Animal Conservation, Northwest University, Xi’an, China; 3 College of Life Sciences, Northwest University, Xi’an, China

## Abstract

Fitness-determining interactions with fungi have often been considered a by-product of social evolution in insects. In higher termites, the mutualistic association between the basidiomycete genus *Termitomyces* and Macrotermitinae is well known. However, whether and how lower termites use fungi is unclear. Here, we found a large amount of brown sclerotium-forming fungi in egg piles of the lower termite *Reticulitermes labralis* and identified the sclerotia as *Fibulorhizoctonia* sp. There was a significant difference in morphology between the sclerotia and the termite eggs. The workers of *R. labralis* and *R. chinensis* actively gathered the sclerotia into the egg piles within their nests, whereas the workers of *R. aculabialis* did not gather sclerotia outside their nests. None of the sclerotia in the egg piles germinated in the presence of workers. However, the sclerotia germinated in the absence of workers, and then the hyphae killed the termite eggs. The data from cellulase activity demonstrated that *Fibulorhizoctonia* sp. was able to exhaustively digest cellulose into glucose.We confirmed for the first time that the workers carrying the sclerotia into the piles of eggs is not due to mistaking the sclerotia for their eggs and that the workers of *R. labralis* may be able to select favourite fungi.

## Introduction

Fungi occupy every natural environment on earth. Fitness-determining interactions with fungi have often been considered a by-product of social evolution in insects^1,2^. Interactions between termites and fungi are found in multiple groups, ranging across both lower and higher termites. In some cases, fungi play a role in termite nutrition, but they can also influence termite survival and caste development. Based on the presence or absence of flagellated protistan symbionts in the hindgut of termites, they are conventionally grouped into lower and higher termites^3^. In higher termites (Termitidae), the mutualistic association between the basidiomycete genus *Termitomyces* and Macrotermitinae is well known. Among termites, the subfamily Macrotermitinae form a monophyletic group of diverse species that originated in Africa and have cultivated specialized fungi (*Termitomyces* spp.) within their nests using woody or litter-based biomass for approximately 31 million years and are able to almost completely decompose lignocelluloses. In the nests of termites, the symbiotic fungi grow on a fungus comb constructed by workers from litter. They are found as mycelia and fungus nodules on the fungus comb surface. Both the fungi and the fungus comb are consumed by the termites^4^. However, whether and how lower termites using fungi is still unclear, and the exact function of fungi is unknown.

Brown sclerotia of fungi were first discovered in egg piles of the lower termite *Reticulitermes speratus* by termitologist Matsuura in Japan^5^. The queens lay eggs in different places in the nests. When workers recognize the scattered eggs, they bring them together and heap them up in order to tend them. Matsuura inferred that the sclerotia mimic termite eggs both morphologically and chemically^5,6^. Is it really ture that the workers aren’t able to distinguish between eggs and sclerotia? In China, we found a large amount of brown sclerotium-forming fungi in egg piles of the lower termite *R. labralis*. These sclerotia can germinate hyphae in a few days and form new sclerotia in culture medium. Our laboratory observation showed that the workers of *R. labralis* actively gathered the sclerotia which were scattered outside nests and carried them into the egg piles. Although we observed that the workers were very interested in sclerotia and treated them as eggs, the purpose of the workers gathering sclerotia and the interactions between them remain unclear.

In this study, we identified the fungus by the analysis of rDNA sequences and identified characteristic morphological features. Second, we compared the morphological differences between the sclerotia and termite eggs (*R. labralis*, *R. aculabialis* and *R. chinensis*) in order to infer whether it was easy for workers to distinguish the sclerotia from the eggs, and then we investigated whether the workers of *R. labralis*, *R. aculabialis* and *R. chinensis* gathered sclerotia from outside the nest by using a sclerotia-carrying test. Finally, the development and cellulase activity of the fungus were investigated.

## Results

### Identification and development of the sclerotia

**Figure 1:**
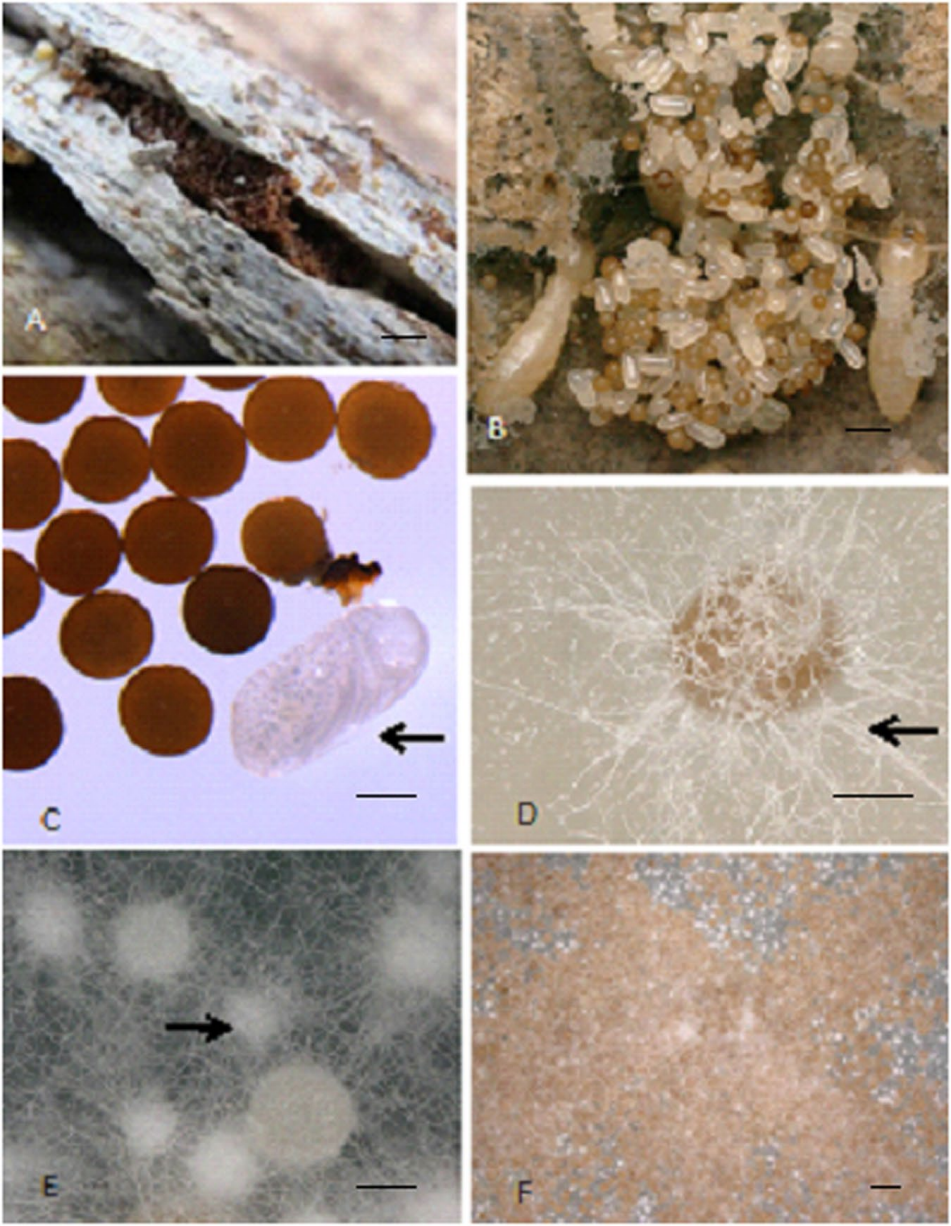
The development of the sclerotia. **(A**) Brown sclerotia were found within the piles of egg in field nests of *R. labralis*. No hyphae were observed on the surface of sclerotia. Scale bars = 1200 μm. (**B**) The sclerotia and eggs were tended by workers of *R. labralis*. Scale bars = 800 μm. (**C**) There was a significant difference in morphology between the eggs( → ) and sclerotia. Scale bars = 200 μm. (**D**)The sclerotia germinated PDA after 4 days. The white hyphae were observed ( → ). Scale bars = 200 μm. (**E**)The white small particles (immature sclerotia, → ) were formed on PDA after 12 days. Scale bars = 200 μm. (**F**) The fungal colonies became brown in color because of the presence of a lot of mature sclerotia with high melanin content after 16 days. Scale bars = 800 μm.

The brown hard balls tended by workers of *R. labralis* were the sclerotia of fungus *Fibulorhizoctonia* sp. affiliated in Atheliaceae (Basidiomycota), (Fig. 1A–C). The sclerotia rRNA sequences, including 18S rRNA segment, ITS1, 5.8S rRNA, ITS2 complete sequence and 28S rRNA segment, were shown below:

5′-GAGGT CAATT GATAT AAAGC TGTCC GAAGA CCATT GTATG CGGCA CCTCG ATCCT AGCCA ACAGC GCAAC ATGAT TATCA CACTG ATGAC CGAAA CGGAC CAAAG TCAAT TCCGC GAATG CATTT GAGAG GAGCC GACCC GGGTG GGGGC CAGCA CGCTC CACAA TCCAA GCCTC CGAAT CGAAA ACAAA TCCGA TCGGG GTTGA GAATT TAATG ACACT CAAAC AGGCA TGCTC CTCGG AATAC CAAGG AGCGC AAGGT GCGTT CAAAG ATTCG ATGAT TCACT GAAAA TCTGC AATTC ACATT ACTTA TCGCA TTTCG CTGCG TTCTT CATCG ATGCG AGAGC CAAGA GATCC GTTGT TGAAA GTTGT ATTAA TTTTT TTATC GACGC TTTTT TAGGG CGAGG ATATA GATTT ACATT CTGAA GACAT ACAGT GTTTA TGATA AACAT GGGAC GACTC CATCA CAGAG CGCCC CACGG TTGGT TCACA GGGGT TGGTT GGTAT TAACG GGCAG AGACG TGCAC ATGCC CCGAG GGGCC AGCAC AGCCA AAGCC TTATA ATTCA ATAAT GATCC TTCCG CAGGT TCACC TACGG AAACC TTGTT-3′

**Figure 2:**
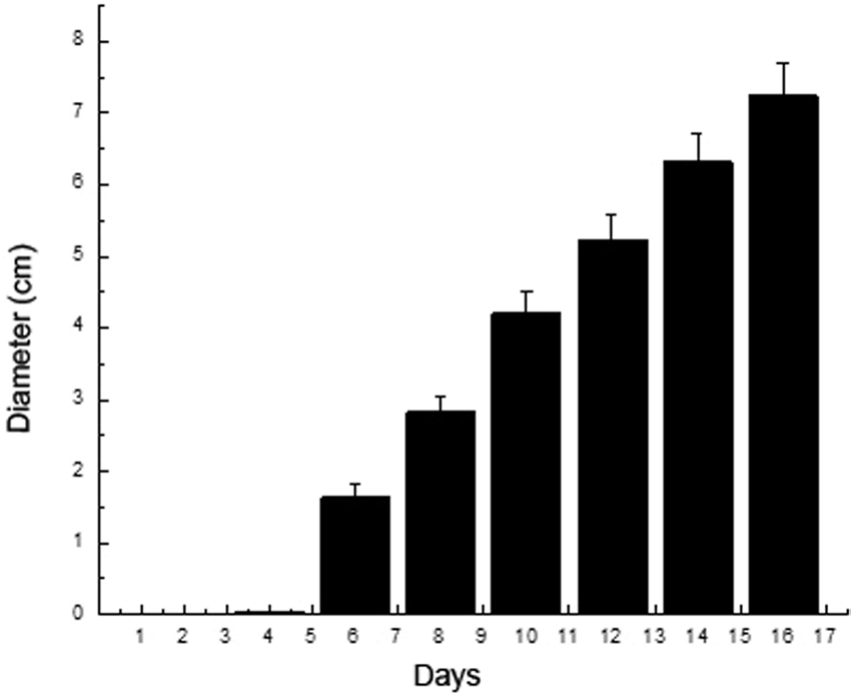
The growth of the fungal colonies in PDA (n = 5). The columns represent the means; bars represent SD.

After the sclerotia were cultured on PDA for 4 days, the white hyphae from the sclerotium germination were observed (Fig. 1D). Hyphae grew and branched to form a filamentous network called a mycelium. The young mycelium was white and cotton-like. The size of the fungal colonies gradually increased over time. After 12 days, the white small particles (immature sclerotia) were formed by a compact mass of interwoven hyphae (Fig. 1E), and the diameter of fungal colonies was 5.23 ± 0.33 cm (Fig. 2). After 16 days, the fungal colonies became brown because of the presence of a large amount of mature sclerotia with a high melanin content (Fig. 1F).

### Workers sclerotia-carrying test

**Figure 3:**
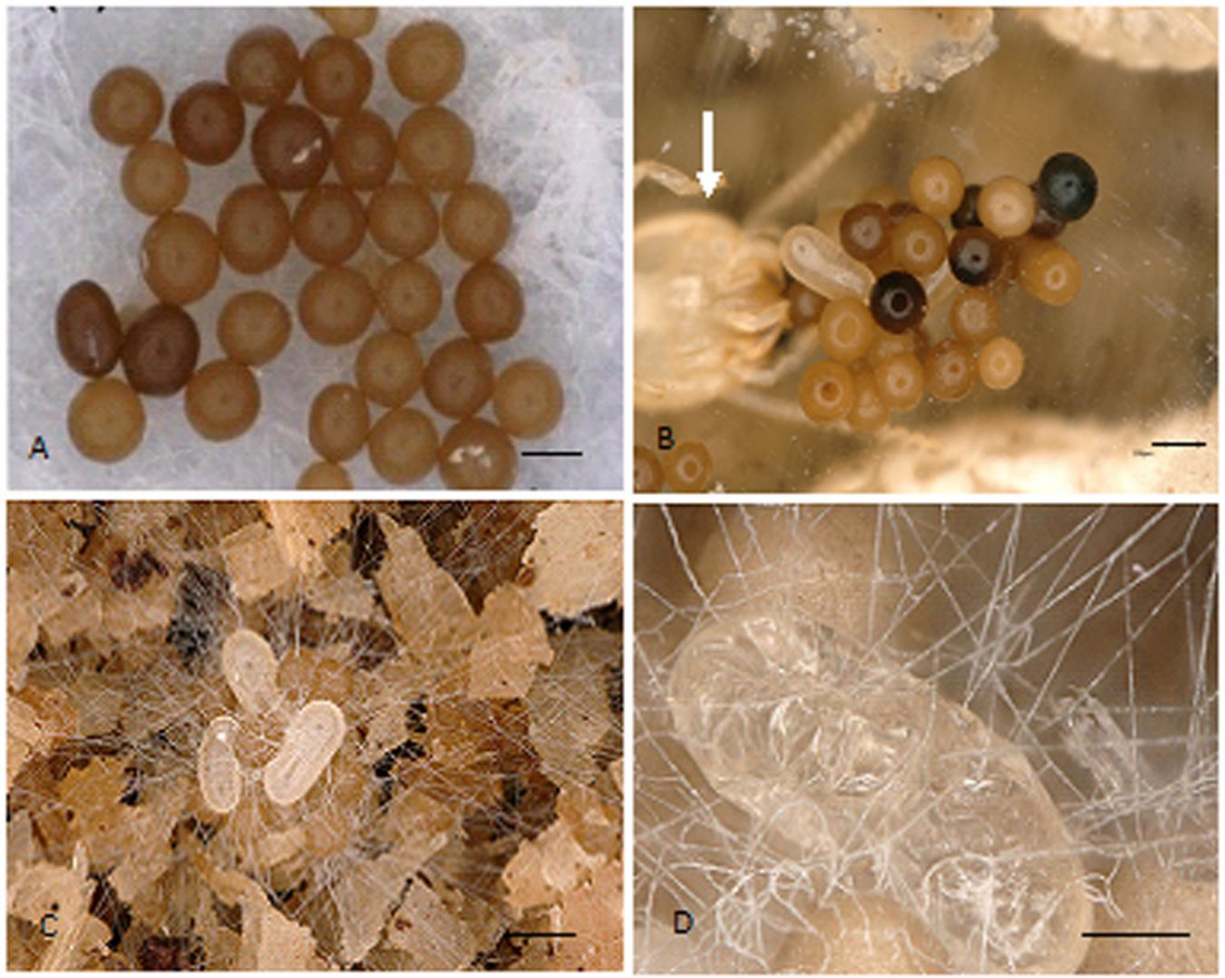
The workers carried the sclerotia into their nests. (**A**) The sclerotia were placed on a filter paper on the nests of *R. labralis*. Scale bars = 300 μm. (**B**) The sclerotia were found in egg piles in the nests of *R*. *labralis* after 24 h. A worker ( → ) was tending the sclerotia and eggs. Aging sclerotia were black. Scale bars = 400 μm. (**C**) The sclerotia in egg piles germinated in the absence of workers. Scale bars = 400 μm. (**D**) The fungal hyphae invaded and killed the termite eggs in the absence of workers.Scale bars = 200 μm.

After 24 h we found that the number of sclerotia on the filter papers outside five nests of *R. labralis* was 0, 0, 0, 0 and 0, respectively. The number of sclerotia on the filter paper outside five nests of *R. chinensis* was 0, 0, 0, 0 and 0, respectively. Moreover, the sclerotia were found in egg piles in nests of *R. labralis* and *R. chinensis*, indicating that the sclerotia were carried by workers into their nests (Fig. 3B). The workers carried the sclerotia to their egg piles in the nursery room, where the eggs hatched into larvae. None of the sclerotia in the egg piles germinated in the presence of workers. However, the sclerotia germinated in the absence of workers, and then the fungal hyphae invaded and killed the termite eggs (Fig. 3C,D). We found that the workers of *R. aculabialis* did not carry sclerotia even if they discovered the sclerotia outside their nest. The number of sclerotia on the filter papers outside five nests of *R. aculabialis* remained at 30.

When one egg was placed in the pile of sclerotia, the workers picked up and carried eggs first, indicating that the sclerotia weren’t recognized as eggs by workers (a video in Supplementary imformation). Our observations showed that the workers carrying the sclerotia into the piles of eggs was not due to mistaking the sclerotia for their eggs.

### Morphological comparison of the sclerotia and termite eggs

**Figure 4:**
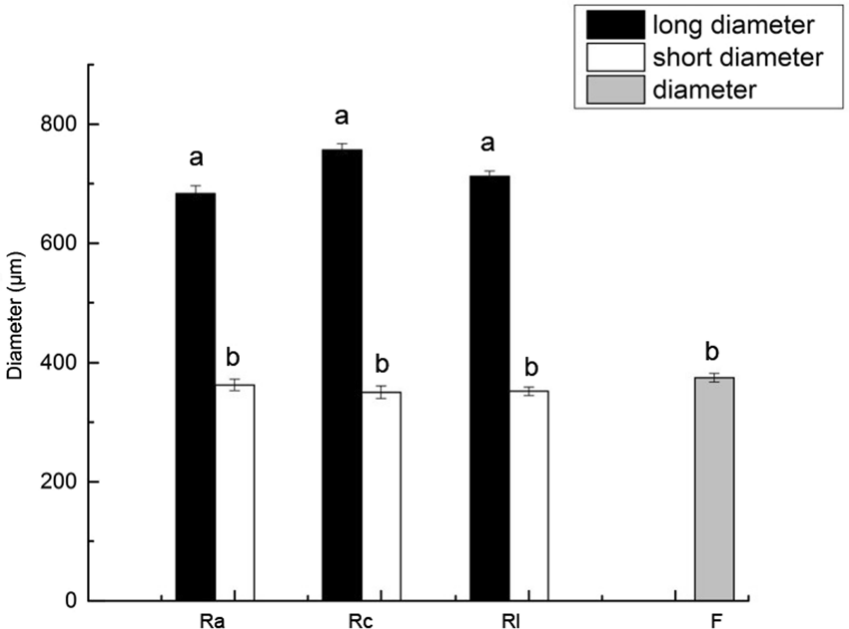
The mean sizes of the sclerotia and eggs of *R. aculabialis*, *R. chinensis* and *R. labralis* (n = 10). The columns represent the means; bars represent SD. The letters (**a**,**b**) above the columns indicate significant differences (p < 0.05). Ra, *R. aculabialis*; Rc, *R. chinensis*; Rl, *R. labralis*; F, the sclerotia of *Fibulorhizoctonia* sp.

The sclerotia were brown, hard and spherical or nearly spherical (0.3–0.4 mm in diameter). The eggs of *R. labralis*, *R. aculabialis* and *R. chinensis* were transparent, soft and oval (Fig. 1C). Although there was no significant difference between the diameter of sclerotia and the short diameter of termite eggs (*p* > 0.05), the long diameter of termite eggs was approximately two-fold longer than the diameter of sclerotia (*p* < 0.05). Therefore, there was a significant difference in morphology between the eggs and sclerotia (Fig. 4).

### Cellulase activity of *Fibulorhizoctonia* sp

**Figure 5:**
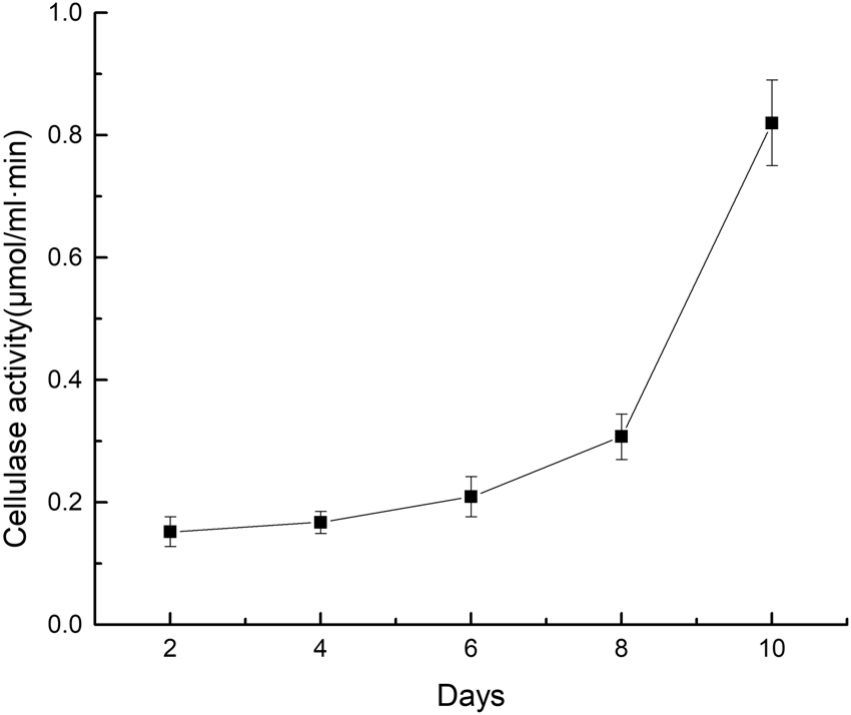
Changes in cellulase activity with culture time. The black spots represent the means; bars represent SD.

The degradation of filter paper is directly related to digestibility of naturally occurring cellulase. With increasing days of cultivation, the cellulase activity also increased. According to the filter paper degrading activity (Fig. 5), cellulase activity remained lower at the beginning of cultivation, with a value of 0.152 ± 0.024 μmol ml^−1^ min^−1^. The cellulase activity increased rapidly after 8 days (0.307 ± 0.036 μmol ml^−1^ min^−1^) and increased (0.820 ± 0.070μmol ml^−1^ min^−1^) after 10 days.

## Discussion

We identified that the brown balls in egg piles of *R. labralis* were the sclerotia of *Fibulorhizoctonia* sp. *Fibulorhizoctonia* is a genus of fungi in the Atheliaceae family, and three widespread species have been named, *F. carotae*, *F. centrifuga and F. psychrophila*, which were consistently isolated from decayed fruits, vegetables and wooden storage bins^1,7,8^. Most fungi produce some type of durable microscopic structure such as a spore that is important for dispersal and/or survival under adverse conditions. However, some fungi also produce dense aggregations of fungal tissue called sclerotia. These persistent structures help fungi survive challenging conditions, such as freezing temperatures, desiccation, microbial attack, or the long-term absence of a host^9,10^. *Fibulorhizoctonia* is known as a decay fungus, while the lower termites like to eat rotten wood infected with fungi. Therefore, we infer that the sclerotia in egg piles of termite *R. labralis* were collected from rotten wood, and the fungus found in egg piles of *R. labralis* may be a new species of *Fibulorhizoctonia*.

Our study revealed that although only one wild colony of *R. labralis* had the sclerotia of *Fibulorhizoctonia* sp., the workers from other colonies of *R. labralis* also actively picked up the sclerotia that were scattered outside the nests and carried them into the nests. This finding indicates that the workers of *R. labralis* are interested in the sclerotia and these sclerotia are an extra gain for the foraging workers outside. *Reticulitermes* are foraging termites. To gain access to food, the workers sooner or later have to forage outside the nest^11^. We considered that when the workers of *R. labralis* left their nest to exploit new food resources, they found the sclerotia and then carried these sclerotia into their nest. Interestingly, in the colonies of *R. labralis*, we did not find the hyphae from sclerotial germination that might kill termite eggs. Therefore, we suggest that there is no interaction between eggs and the sclerotia when they are under the care of workers.

We confirmed for the first time that the workers carrying the sclerotia into the piles of eggs is not due to mistaking the sclerotia for their eggs.There was a significant difference in morphology between the sclerotia and the termite eggs, indicating that the workers were able to discriminate between the sclerotia and eggs. The long diameter of the eggs was approximately 2-fold longer than the diameter of sclerotia, and in contrast to the eggs, the sclerotia were every hard. Almost all worker termites are blind, as they lack compound eyes. However, the major sensory field of most insects are the antennae, mouthparts, leg, wings, genitalia, cerci, and the ovipositor, which possess the mechanoreceptive senses, including tactile (touch), proprioceptive and sound or vibration. Moreover, the two volatile chemicals (n-butyl-n-butyrate and 2-methyl-1-butanol) in the queen pheromone are also emitted by eggs of *R. speratus* and an antibacterial protein lysozyme, which also functions as an egg-recognition signal, is synthesized in eggs^12,13^. These compounds indicate egg presence and function as an orientation pheromone guiding workers to care for eggs^14^. Therefore, there are fundamental differences in morphology, chemistry and physical properties (for example, hardness and vibration) between the eggs and sclerotia. We found that the workers picked up and carried the egg first when one egg was placed in the pile of sclerotia. We believe that the workers carrying the sclerotia to the piles of eggs is not due to mistaking the sclerotia for their eggs.

Our study revealed that not all of the *Reticulitermes* species collected the sclerotia and were interested in the sclerotia. We found that the workers of *R. labralis* and *R. chinensis* carried the sclerotia into their egg piles in nests, whereas the workers of *R. aculabialis* did not carry sclerotia when they discovered the sclerotia outside the nests. To date, the sclerotia of *Fibulorhizoctonia* sp. have only been found in four *Reticulitermes* termites in Japan *(R. speratus*, *R. kanmonensis*, *R. amamianus*, and *R. miyatakei*) and four species in the United States (*R. flavipes*, *R. virginicus*, *R. hageni*, and *R. malletei*)^15^. Interestingly, in our study, although the sclerotia were only found in a natural colony of *R. labralis* and interactions between them were not obligate and therefore not symbiotic, the workers of *R. labralis* and *R. chinensis* from laboratory colonies also actively gathered the sclerotia into their nests which was not observed in *R. aculabialis*. This variation is an important sign that there are significant differences in the ability to recognize and respond to the sclerotia of *Fibulorhizoctonia* sp. among *Reticulitermes* species.

β-glucosidase is known as a multifunctional enzyme for social maintenance in terms of both cellulose digestion and social communication in termites^16^.The data from cellulase activity demonstrated that the fungus *Fibulorhizoctonia* sp. was able to exhaustively digest cellulose into glucose, indicating that the *Fibulorhizoctonia* sp. had “complete” cellulases. The fungus is the main decomposer of lignocellulose, the predominant component of (mostly) dead plant material. Lignocellulose consists of cellulose (20–50%), hemicelluloses (15–35%) and lignin (18–35%). Cellulose, a linear polysaccharide consisting of β-1,4-linked D-glucopyranosyl units, is the major component of plant material and the most abundant biomass on earth^3^. Enzymes that effect hydrolysis of cellulose into glucose are known as cellulases. Complete cellulases typically consist of endo-β-1,4-glucanases, cellobiohydrolases and β-glucosidases, which convert the short-chain sugars into glucose. Moreover, β-glucosidases have been found in egg surface of *R. speratus*^17^. These study results indicate that the workers of *Reticulitermes* may be able to select favourite fungi by recognizing β-glucosidases. Therefore, we infer that the main functions of the sclerotia in the egg piles of *R. labralis* fall into two categories: (i) Hyphae from sclerotial germination are an additional food source for termites. We did not observe sclerotial germination in the egg piles of *R. labralis*, probably because the termites had eaten up all hyphae. (ii) The sclerotia provision cellulases to work synergistically and/or complementarily with endogenous enzymes of termite. Further information aboult the distribution of the fungus *Fibulorhizoctonia* sp. on a global scale is required in order to elucidate the evolutionary background of the *Reticulitermes- Fibulorhizoctonia* relationship^5^.

## Materials and Methods

### Development of the sclerotia

Brown sclerotia were found in the piles of egg within nests of *R. labralis* in Xi’an, China, in May 2014 (Fig. 1A). The sclerotia were soaked in a solution of sodium hypochlorite (approximately 1% active chlorine) for 10 min, this step was repeated three times, and then the sclerotia were rinsed with sterilized distilled water three times. Five surface-sterilized sclerotia were cultured in five Petri dishes containing potato dextrose agar (PDA) at 27 °C for 20 days. The sclerotia germination, the production of new sclerotia, and the diameter of the fungal colonies were recorded every two days using VHX-5000 Digital Microscope (Keyence Corporation, Osaka, Japan).

### Identification of the sclerotia

The sterilized sclerotia were cultured on PDA in test tubes until sclerotia germination for DNA extraction. We identified the fungus using morphological and molecular tools, based on sequence analysis of the internal transcribed spacer (ITS) of the rRNA. The entire ITS1-5.8S-ITS2 region was amplified and sequenced using the universal fungal primers ITS1 (5′-TCCGTAGGTGAACCTGCGG-3′) and ITS4 (5′-TCCTCCGCTTATTGATATGC-3′)^17^. Identification of the sclerotia was performed by the Institute of Microbiology of the Chinese Academy of Sciences (IMCAS). IMCAS is the largest microbiological research institution in China.

### Sclerotia-carrying test

To test whether termite workers gathered sclerotia from outside the nest, we conducted a sclerotia-carrying bioassay. *R. labralis* and *R. aculabialis* were collected in Xi’an, China, and *R. chinensis* was collected in Chengdu, China. Thirty sclerotia were placed on a moist filter paper (Fig. 3A), and then the filter papers with thirty sclerotia were put on nests of *R. labralis*, *R. aculabialis* and *R. chinensis*. Five replications were made. The colonies of *R. labralis*, *R. aculabialis* and *R. chinensis* without the sclerotia were established in Petri dishes with sawdust under laboratory conditions for one year before the sclerotia were put on their nests. The sclerotia on the filter papers were counted after 24 h, and we observed whether there were sclerotia in the termite nests.

To confirmed whether the workers carrying the sclerotia into the piles of eggs was due to mistaking the sclerotia for their eggs, one egg of *R. labralis* and ten sclerotia together were placed in a Petri dish, and then added the workers of *R. labralis*. Five replications were made. We recorded the behavior of workers using VHX-5000 Digital Microscope (Keyence Corporation, Osaka, Japan).

### Morphological comparison of the sclerotia and termite eggs

To determine whether the workers mistook the sclerotia for their eggs, we identified the difference in morphology between the sclerotia and eggs. The size of sclerotia and eggs of *R. labralis*, *R. aculabialis* and *R. chinensis* was measured using a digital microscope. Measurements of each sclerotia were performed using two perpendicular diameters of the sclerotia, and measurements of each eggs were performed using the long diameter and short diameter of the eggs. All values were expressed as the means ± SD. The significant differences were identified with the non-parametric Kruskal-Wallis test followed by Dunn’s multiple comparisons test. *P* < 0.05 was considered statistically significant.

### Cellulase activity of the fungi

Four hundred sclerotia from the piles of egg in the nests of *R. labralis* were sterilized and then cultured in Vogel’s Medium N at 120 rpm at 28 °C for 10 days. During this period, 1 ml of the fermentation product was collected every two days and then centrifuged at 8000 rpm for 10 min. After high-temperature sterilization filter paper (Whatman No. 1 (1 cm × 1 cm)) was put into tubes with 0.2 ml sodium acetate buffer (SAB), and the supernatant (0.2 ml) was incubated with the filter paper at 50 °C for 1 h. Water was removed, and 1 ml of 3,5-dinitrosalicylic acid (DNS) was added. The DNS reagent was prepared by dissolving 6.3 g DNS (Kermel, China) in 500 ml of distilled water and adding 262 ml 2 mol L^−1^ NaOH (Kermel, China). After complete dissolution, 185 g Na-K tartrate (Kermel, China), 5 g crystalline phenol (Kermel, China), and 5 g sodium sulphate anhydrous (Kermel, China) were added, and the volume was filled to 1 L. The sample was placed in a boiling water bath for 10 min. The tubes were then cooled to room temperature rapidly. Lastly, 2 ml SAB was added to the tubes, and then 20 min was allowed to elapse. The mixture was detected colorimetrically with UV-2550 spectrophotometer (Shimadzu, Japan) at 540 nm, using glucose as a standard. The same volume of SAB was used as the control. One unit (U) of enzyme activity was defined as the amount of enzyme capable of releasing one μmol reducing sugar per minute. The enzyme assays were repeated three times. A standard curve prepared with glucose (Kermel, China) in the range from 0 to 1.0 g L^−1^.

## Supplementary information

Supplementary Information

## References

[1] Vries RP, Lange ES, Wosten HAB, Stalpers JA (2008). Control and possible applications of a novel carrot-spoilage basidiomycete. Fibulorhizoctonia psychrophila. Antonie van Leeuwenhoek.

[2] Biedermann PHW, Rohlfs M (2017). Evolutionary feedbacks between insect sociality and microbial management. Current Opinion in Insect Science.

[3] Ni J, Tokuda G (2013). Lignocellulose-degrading enzymes from termites and their symbiotic microbiota. Biotechnology Advances.

[4] Li H (2017). Lignocellulose pretreatment in a fungus-cultivation termite. Proc Natil Acad Sci USA.

[5] Matsuura K, Tanaka C, Nishida T (2000). Symbiosis of a termite and a sclerotium-forming fungus:sclerotia mimic termite eggs. Ecological Research.

[6] Matsuura K, Yashiro T, Shimizu K, Tatsumi S, Tamura T (2009). Cuckoo fungus mimics termite eggs by Producing the Cellulose-Digesting Enzyme beta-Glucosidase. Current Biology.

[7] Hermansen A, Wanner L, Nærstad R, Klemsdal SS (2012). Detection and prediction of post harvest carrot diseases. Eur J Plant Pathol..

[8] Wenneker M (2016). *Fibulorhizoctonia psychrophila* is the causal agent of lenticel spot on apple and pear fruit in the Netherlands. Eur J Plant Pathol.

[9] Stajich JE (2009). The Fungi. Current Biology.

[10] Smith ME, Henkel TW, Rollins JA (2015). How many fungi make sclerotia? Fungal. Ecology.

[11] Korb J, Hartfelder K (2008). Life history and development-a framework for understanding developmental plasticity in lower termites. Biol. Rev..

[12] Matsuura, K., Tamura,T., Kobayashi, N., Yashiro, T. & Tatsumi, S. The antibacterial protein lysozyme identified as the termite egg recognition pheromone. *PLoS One*, e813, 10.1371/journal.pone.0000813 (2007). 10.1371/journal.pone.0000813 PMC1950569 17726543

[13] Matsuura K (2010). Identification of a pheromone regulating caste differentiation in termites. Proc Natil Acad Sci USA.

[14] Matsuura K (2012). Multifunctional queen pheromone and maintenance of reproductive harmony in termite colonies. J Chem Ecol..

[15] Matsuura K (2005). Distribution of termite egg-mimicking fungi (“termite balls”) In *Reticulitermes* spp. (Isoptera: Rhinotermitidae) nests in Japan and the United States. Appl. Entomol. Zool..

[16] Shimada K, Maekawa K (2014). Gene expression and molecular phylogenetic analyses of beta-glucosidase in the termite *Reticulitermes speratus* (Isoptera: Rhinotermitidae). Journal of Insect Physiology.

[17] White, T. J., Bruns, T., Lee, S. & Taylor, J. Amplification and direct sequencing of fungal ribosomal RNA genes for phylogenetics. In: Innis, M. A., Gelfand, D. H., Sninsky, J. J., White, T. J. (Eds), PCR Protocols: a guide to methods and applications. Academic Press, New York, pp. 315–322 (1990).

